# Right Scapular Swelling Revealed to Be a Spontaneous Lateral Chest Wall Hematoma: An Intriguing Case Report

**DOI:** 10.7759/cureus.10151

**Published:** 2020-08-31

**Authors:** Muhammad Saad Choudhry, Anum Sultan, Maria Hassan, Muhammad Ali, Syed Muhammad Hussain Zaidi

**Affiliations:** 1 General Surgery, Civil Hospital Karachi, Dow University of Health Sciences, Karachi, PAK; 2 Radiology, Dr. Ziauddin Hospital, Karachi, PAK; 3 Internal Medicine, Civil Hospital Karachi, Dow University of Health Sciences, Karachi, PAK

**Keywords:** spontaneous, chest wall, hematoma, intercostal artery, anticoagulant

## Abstract

Chest wall hematoma commonly occurs as a result of blunt thoracic trauma. We report an intriguing case of spontaneous lateral chest wall hematoma that presented with right scapular swelling and tenderness on palpation having hemodynamic instability without any prior history of recent trauma or surgery. Chest x-ray and ultrasound were carried out at the time of admission to evaluate the swelling, followed by contrast-enhanced CT (CECT) of the chest to identify and localize the bleeder. Transcatheter arterial embolization (TAE) of the intercostal artery was performed to treat the condition successfully. CECT is found to be vitally important in localizing bleeders. Other possible options include exploratory thoracotomy, video-assisted thoracic surgery (VATS) exploration, and angiography.

## Introduction

Chest wall hematoma is a relatively rare condition that is often under-reported in medical literature. Although it commonly develops secondary to trauma, non-traumatic causes may include tumors and anticoagulants [1,2]. It can be described as an accumulation of blood in the extrathoracic space that manifests as a crescent-shaped opacity with its inner border directed against adjacent part of the chest wall on radiography. Chest wall hematomas develop within dense tissues, limiting extensive bleeding by compressing the bleeding vessel and making their detection challenging. On the other hand, hemothorax develops within the pleural cavity, a relatively free space that allows bleeding and is easily detected. The associated early mortality rate is reported to be 2.9% [3].

## Case presentation

Our patient was a 63-year-old male, a previously diagnosed case of degenerative cervical disorder, hypertension, and alcoholism. He underwent an aortic valve replacement surgery in 2011 and was on warfarin since then, maintaining a target international normalized ratio (INR) of 2.5-3.5 (normal range: 0.8-1.1). The patient presented with sudden onset right scapular swelling, which was tender on palpation. An ultrasound of the swelling revealed a well-defined heterogeneous mass in the right posterior chest wall having a depth of around 2.3 cm. No definite vascularity was observed, and swelling was labeled as a hematoma/collection. The patient started to sweat profusely and was rushed to the emergency room immediately because he lost consciousness but eventually regained it on the way. His vitals displayed a picture of hypotensive episode secondary to suspected hemorrhagic shock and was administered intravenous fluids and atropine. Hematological investigations revealed moderate normocytic anemia with hemoglobin of 9.0 g/dL (normal in males: 13.5-17.5 g/dL), hematocrit of 26% (normal in males: 38.3%-48.6%), and platelet count of 155,000/mcL (normal: 150,000-450,000/mcL). Prothrombin time and INR were elevated at 32.8 seconds (normal: 10-13 seconds) and 2.9 (normal: 0.8-1.1), respectively, while partial thromboplastin time was at the normal upper limit, 35.5 seconds (normal: 25-35 seconds). Laboratory tests revealed serum urea 30 mg/dL (normal: 17-49 mg/dL) and creatinine level 0.79 mg/dL (normal in males: 0.9-1.3 mg/dL). Serum electrolytes were as follows: sodium 140 mEq/L (normal: 136-149 mEq/L), potassium 4.1 mEq/L (normal: 3.8-5.2 mEq/L), chloride 107 mEq/L (normal: 98-107 mEq/L), and bicarbonate 25.5 mEq/L (normal: 23-29 mEq/L). C-reactive protein was 3.5 mg/L (normal < 10 mg/L) and liver function tests were alanine transaminase 40 IU/L (normal: 0-45 IU/L) and aspartate transaminase 60 IU/L (normal: 0-35 IU/L). Serum N-terminal pro b-type natriuretic peptide (NT-pro-BNP) was 69 pg/mL (normal < 125 pg/mL) indicating absence of possible heart failure.

**Figure 1:**
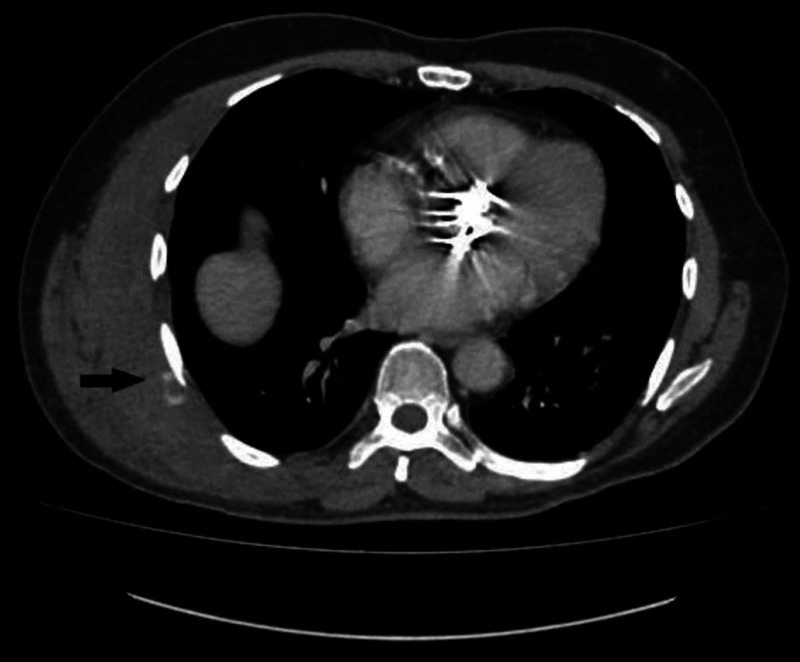
Contrast-enhanced CT chest demonstrates a large chest wall hematoma on the right lateral chest wall. The arrow in black highlights the active bleeder.

Once his vitals became stable, he was scheduled for a contrast-enhanced CT (CECT) chest to localize the bleeding points. During the arterial phase, the scan displayed that no active arterial bleed was present within the hematoma in the right lateral chest wall. However, delayed phase images revealed focal extravasation of contrast within the hematoma in close proximity of the right seventh rib (as displayed by the arrow in Figure 1). The bleeding point was deduced to originate from a tiny branch of the intercostal artery in the right seventh intercostal space. A large acute hematoma was identified in the right upper and mid-lateral chest wall measuring 13.1 x 3.2 cm (anteroposterior x transverse) (Figure 1). There was no evidence of aortic dissection or mediastinal hematoma and no large mass, cavitation, consolidation, or pleural effusion was seen. 

**Figure 2:**
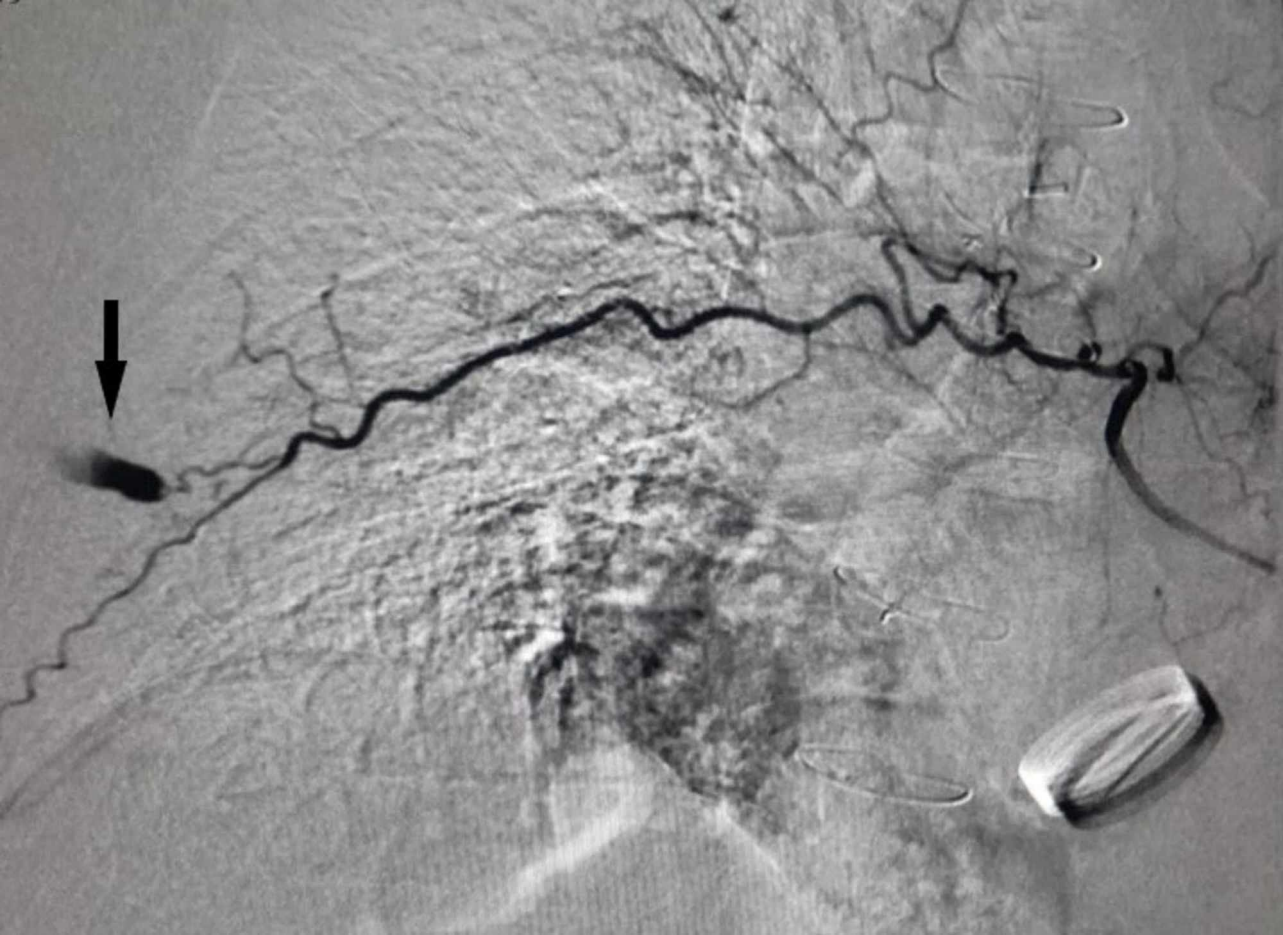
Transcatheter arterial angiography image pre-embolization demonstrating the actively bleeding intercostal artery (arrow in black).

The patient was taken to an angiography suite where emergency transcatheter right intercostal artery angiography and embolization were carried out using a 5 French (Fr) vascular sheath placed in the right common femoral artery and a 5 Fr C2 catheter. On angiography, the active bleeder was identified as a small branch of the intercostal artery in the right seventh intercostal space (contrast extravasation from active bleeder as shown by the arrow in Figure 2). The aortic prosthetic valve was also noted.

**Figure 3:**
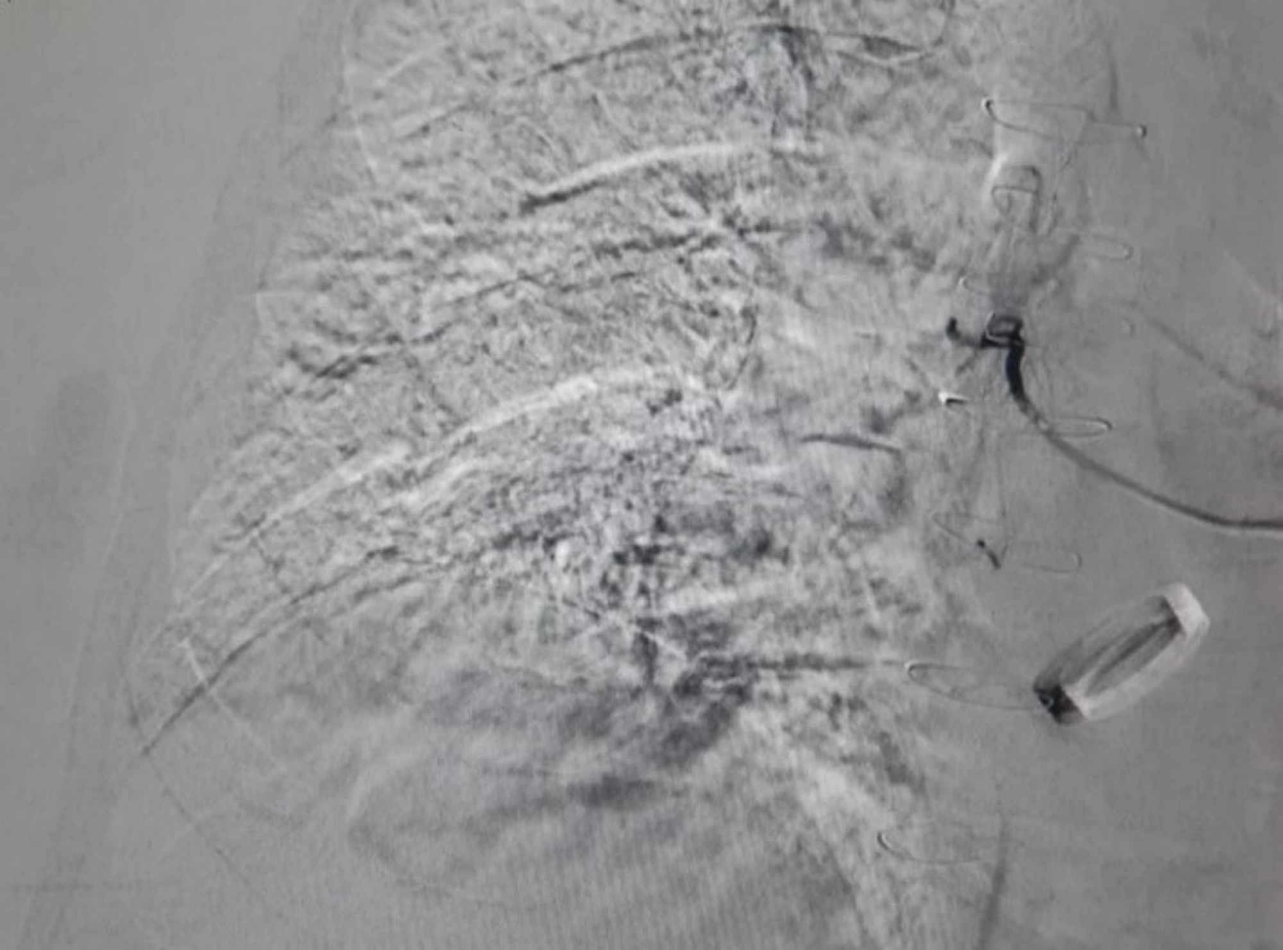
Transcatheter arterial angiography image post-embolization demonstrating the bleeding vessel selectively embolized.

Subsequently, the vessel was selectively cannulated using a microcatheter, and embolization was performed using 150-250 microns polyvinyl acetate (PVA) particles and two 10-mm pushable microcoils. Hence, complete occlusion of the abnormal actively bleeding vasculature was achieved (demonstrated by the absence of contrast extravasation in Figure 3). The patient was shifted to the intensive care unit, where blood products were transfused. He was kept under strict monitoring of vitals and INR because anti-hypertensives and warfarin were put on hold due to hemodynamic instability. 

## Discussion

Chest wall hematoma can be defined as a collection of blood in the extrathoracic space, i.e., within the chest wall's dense tissues. Radiological images of chest wall hematomas produce a characteristic crescent shape, with its outer border directed against the chest wall tissues and the inner border limited by the neighboring part of the rigid chest wall. They are usually self-limited and do not expand rapidly because of the tightly packed chest wall tissue that limits hematoma development by compressing the active bleeder(s). However, hematoma formation within a restricted space can compress adjacent organs to cause hemodynamic instability creating a state of a medical emergency. Celik et al. reported a similar case of spontaneous extrathoracic lateral chest wall hematoma in an elderly male. The patient was reported to be on oral warfarin therapy and had no history of prior trauma or surgery, analogous to our case presented herein. The hematoma was surgically removed through a submammary incision, unlike our patient, who underwent transcatheter arterial embolization (TAE) to treat the condition [4]. 

Chest pathologies like pneumothorax or hemothorax can be promptly detected on elementary chest radiography, but chest wall hematoma may be easily overlooked. However, CT is a sensitive modality that can detect pulmonary contusions and chest wall hematomas [5]. CECT can localize bleeding vessels even when a patient is hemodynamically unstable. Proper identification of bleeder(s) can help in accurate angiographic embolization to achieve hemostasis [6,7]. Other potential options, including exploratory thoracotomy, video-assisted thoracoscopic surgery (VATS), and angiography, can be performed in the absence of coagulopathy when CECT results are negative [8]. TAE is often employed to manage active arterial bleed from splenic, hepatic, and renal parenchymal injury. It can also be used to manage pelvic fractures in trauma patients [9]. In our case, TAE was performed, as it was the most effective and minimally invasive option available, considering our patient's coagulation profile, which did not qualify him as a suitable candidate for exploratory surgery or VATS.

## Conclusions

Spontaneous extrathoracic chest wall hematoma is an extremely rare condition. The diagnosis and localization of bleeding vessel(s) in patients who are actively bleeding and hemodynamically unstable can be performed using CECT, an exceptionally useful modality. TAE is a reliable and minimally invasive technique that can be effectively employed in cases having deranged coagulation parameters and hemodynamic instability to successfully treat the condition. Other conventional and possible options like exploratory thoracotomy and VATS can be used in patients who are hemodynamically stable and have normal coagulation parameters.
